# Protective role of hesperidin against γ-radiation-induced oxidative stress and apoptosis in rat testis

**DOI:** 10.1186/s40709-017-0059-x

**Published:** 2017-03-01

**Authors:** Nadia Z. Shaban, Ahmed M. Ahmed Zahran, Fatma H. El-Rashidy, Ahmad S. Abdo Kodous

**Affiliations:** 1 Biochemistry Department, Faculty of Science, Alexandria University, Alexandria, Egypt; 2 Radiation Biology Department, National Center for Radiation Research and Technology (NCRRT), Egyptian Atomic Energy Authority, Cairo, Egypt

**Keywords:** Hesperidin, Oxidative stress, Apoptosis, Testis injury, Gamma radiation, Protection

## Abstract

**Background:**

Gamma (γ) ray, an electromagnetic radiation, is occasionally accompanying the emission of an alpha or beta particle. Exposure to such radiation can cause cellular changes such as mutations, chromosome aberration and cellular damage which depend upon the total amount of energy, duration of exposure and the dose. Ionizing radiation can impair spermatogenesis and can cause mutations in germ cells. In general, type B spermatogonia are sensitive to this type of radiation. The current study was carried out to evaluate the protective role of hesperidin (H), as a polyphenolic compound, on rat testis injury induced by γ-radiation.

**Methods:**

Rats were divided into groups including C group (control rats), R (irradiated) group (rats irradiated with γ-radiation), Vehicle (V) group (rats administered with dimethylsulfoxide “DMSO”), H group (rats administered with H only), HR and RH groups (rats treated with H before and after exposure to γ-radiation, respectively). Malondialdehyde (MDA: the end product of lipid peroxidation “LPO”) and xanthine oxidase (XO: it generates reactive oxygen species “ROS”) in testes homogenate as well as nitric oxide (NO: as ROS) in mitochondrial matrix were determined. The apoptotic markers including DNA-fragmentation (DNAF) in testes homogenate and calcium ions (Ca^2+^) in mitochondrial matrix were determined. Superoxide dismutase (SOD) and catalase (CAT) activities in testes homogenate, while reduced glutathione “GSH” in nuclear matrix were determined. Also histopathological examination for testes tissues through electron microscope was studied.

**Results:**

Exposure of rats to γ-radiation (R group) increased the levels of MDA, NO, DNAF, Ca^2+^ and XO activity, while it decreased GSH level, SOD and CAT activities as compared to the C groups; γ-radiation increased oxidative stress (OS), LPO, apoptosis and induced testes injuries. These results are in agreement with the histopathological examination. In contrast, treatment with H before or after exposure to γ-radiation (HR and RH groups, respectively) decreased the levels of MDA, NO, DNAF and Ca^2+^ but increased GSH level and the activities of SOD, CAT and XO as compared to R group and this indicates that H decreased OS, LPO and apoptosis. Also, the histopathological results showed that H improved testis architecture and this is related to the antioxidant and anti-apoptotic activities of H contents. Protection is more effective when H is given before rather than after exposure. Finally, administration of H to healthy rats for a short period had no adverse affect on testes cells.

**Conclusion:**

Hesperidin showed antioxidant and anti-apoptotic activities. It has a protective role against OS, injury and apoptosis induced by γ-radiation in testes. Protection is more effective when H is given before rather than after exposure.

**Graphical Abstract:**
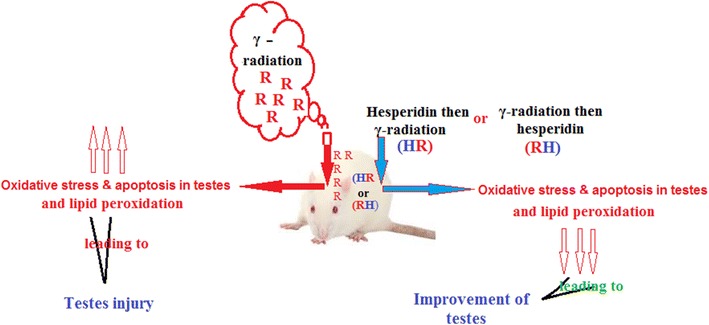
.

## Background

IR has many beneficial applications in medicine, industry and agriculture; it causes changes in the chemical balance of cells by direct and indirect actions. It may cause malignant changes and damage DNA leading to harmful genetic mutations that can be passed on to future generations [1]. In the direct action, IR generates ROS such as superoxide anions (${\text{O}}_{2}^{ - \cdot }$), hydrogen peroxide (H_2_O_2_), and hydroxyl radicals (${}^{ \cdot }{\text{OH}}$), which show high reactivity to a variety of cellular macromolecules [2]. Indirectly, radiation splits water molecules since the radiolytic products are highly reactive and more damaging to biomolecules [3]. On the other hand, SOD catalyzes the reduction of ${\text{O}}_{2}^{ - \cdot }$ to H_2_O_2_ which in turn is broken down by CAT to O_2_ and H_2_O or by glutathione peroxidase (GPx) in presence of GSH to 2 H_2_O. Oxidative stress (OS) emerges when the production of ROS exceeds the capacity of cellular antioxidant defenses [4–8].

**Fig. 1:**
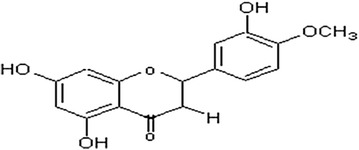
Structure of hesperidin

Hesperidin (H) is a polyphenolic compound (Fig. 1) found in citrus fruits and vegetables as well as in food products and beverages derived from plant, such as tea and olive oil [9]. It is the predominant flavonoid in lemons and oranges while the peel and membranous parts have the highest concentrations. H, in combination with a flavone glycoside called diosmin, is used in Europe for the treatment of venous insufficiency and hemorrhoids [9].

A deficiency of H in the diet has been linked with abnormal capillary leakiness as well as pain in the extremities causing aches, weakness and night leg cramps. H has multiple biological activities such as reduction of capillary fragility, associated with scurvy, antilipemic activities and anti-inflammatory mediator and suppresses cyclooxygenase-2 (COX-2) gene expression. Both H and its aglycone hesperitin have been reported to possess a wide range of pharmacological properties [10]. Therefore, the present study was carried out to investigate the role of H in minimizing the testis damage induced by γ-radiation in a total dose of 8 Gy. The study focused on the determination of apoptotic markers (DNAF in testes homogenate and calcium ions in mitochondrial matrix). Also, OS markers including ROS [such as MDA and XO in testis homogenate beside nitric oxide (NO) in mitochondrial matrix] and cellular antioxidant defenses as GSH and the activities of SOD and CAT were determined in testis homogenate. In addition lipid profile and total protein (TP) as well as electron micrograph of testis were determined.

## Results

### Effect of different doses of γ-radiation on testicular DNAF and ultrastructure configuration

**Fig. 2:**
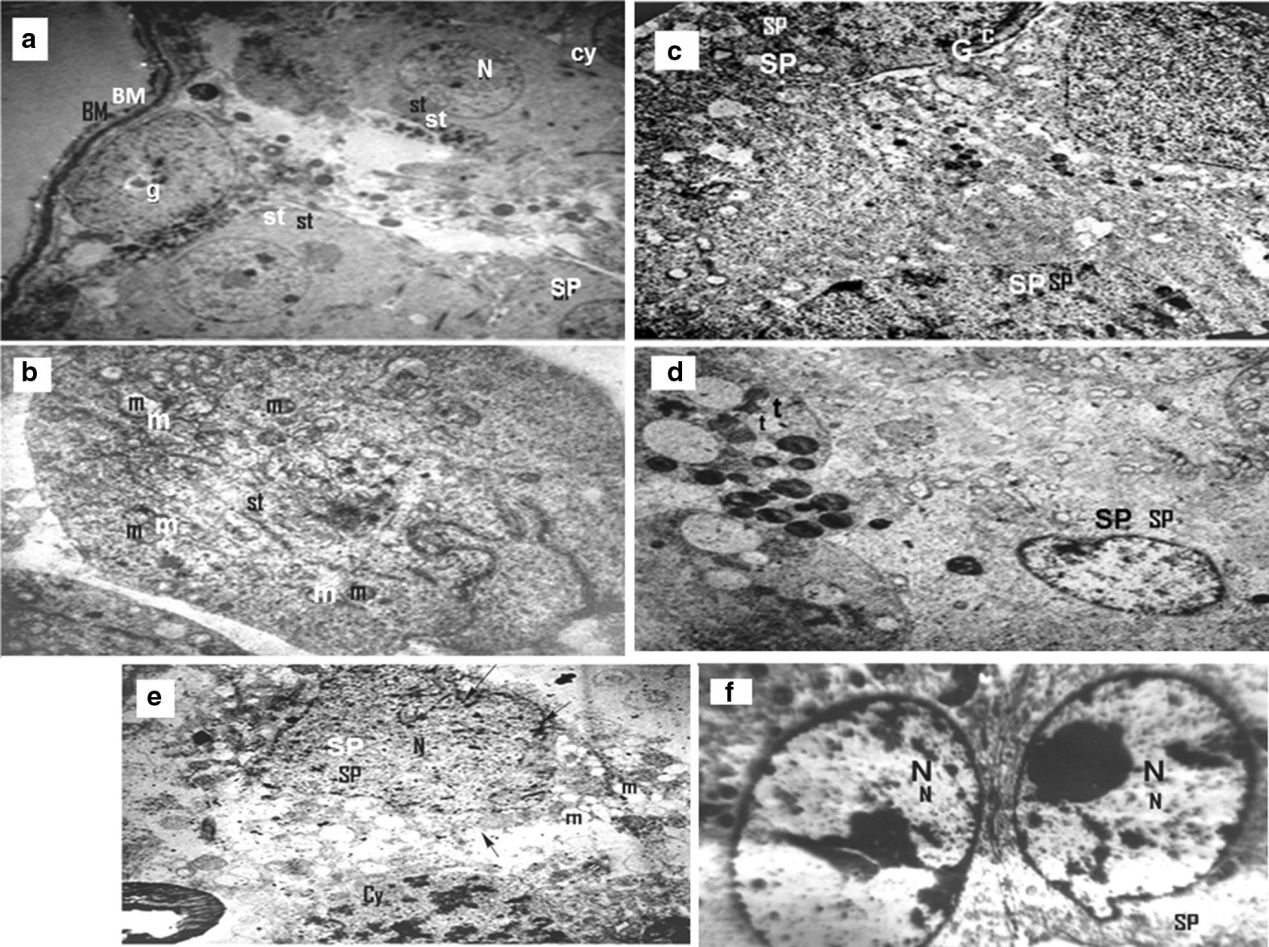
Microscopic examination of testes tissues of rats irradiated with different doses of γ-radiation. The C group **a** showing the thin basement membrane (BM) surrounding the seminiferous tubule. Sertoli cells (st) with indented euchromatic nuclei (N), Spermatid (sp) and Spermatocyte (cy) were seen. Spermatogonia (g) appear with ovoid nucleus (×6000). Rats irradiated with a single dose 2 Gy of γ-radiation **b** showing degeneration of sertoli cells (st) which contain swelling mitochondria (m) (×8000). Rats irradiated with a 4 Gy of γ-radiation **c** showing degeneration of spermatids, (SP) and cluster of spermatids with a characterized chromosomal “cap c” (×8000). Irradiated rats with a total dose 6 Gy **d** showing degenerated spermatids, (SP) and cytoplasmic tags (t) (×8000). Rats irradiated with a total dose 8 Gy of γ-radiation **e** showing highly degenerated spermatids, (SP) with deteriorated cytoplasm and blebbing of nuclear membrane (*arrow*), mitochondria appear as empty vesicles and spermatocytes (cy) with nuclei contain clumps of heterochromatin (×8000). Rats irradiated with a total dose 10 Gy **f** showing highly degenerated spermatids, (SP) with abnormal nuclei (N) which contain condensed heterochromatin (×8000). Number of rats in each group was 5

The results showed that exposure of rats to γ-radiation in doses of 4, 6, 8 and 10 Gy, caused significant increases (*p* < 0.05) in DNAF by about 41.87, 85.12, 182.34 and 184.33%, respectively. These results showed that there were no significant differences in DNAF when compared exposure to 8 Gy and 10 Gy (*p* > 0.05). Also, 2 Gy showed non-significant increase in DNAF level (*p* > 0.05) by about 10.12% as compared to the control. The histological examination through electron microscopy showed that the control rats appeared normal with no changes in the ultrastructure configuration (Fig. 2a). However, the irradiated rats with a single dose of 2 Gy showed degeneration of sertoli cells that contain swelling mitochondria (Fig. 2b). Exposure to 4 Gy dose showed degeneration of spermatids and cluster of spermatids with a characterized chromosomal “cap” (Fig. 2c). Also, 6 Gy dose revealed degeneration of spermatids and cytoplasmic tags (Fig. 2d). Both doses of 8 and 10 Gy showed highly degenerated spermatids, with deteriorated cytoplasm and blebbing of nuclear membrane, mitochondria appeared as empty vesicles and spermatocytes with nuclei contain clumps of heterochromatin (Fig. 2e, f). This indicated that 8 and 10 Gy gave similar effects, so we used 8 Gy in the main experiment.

### Effect of γ-radiation (8 Gy)

**Fig. 3:**
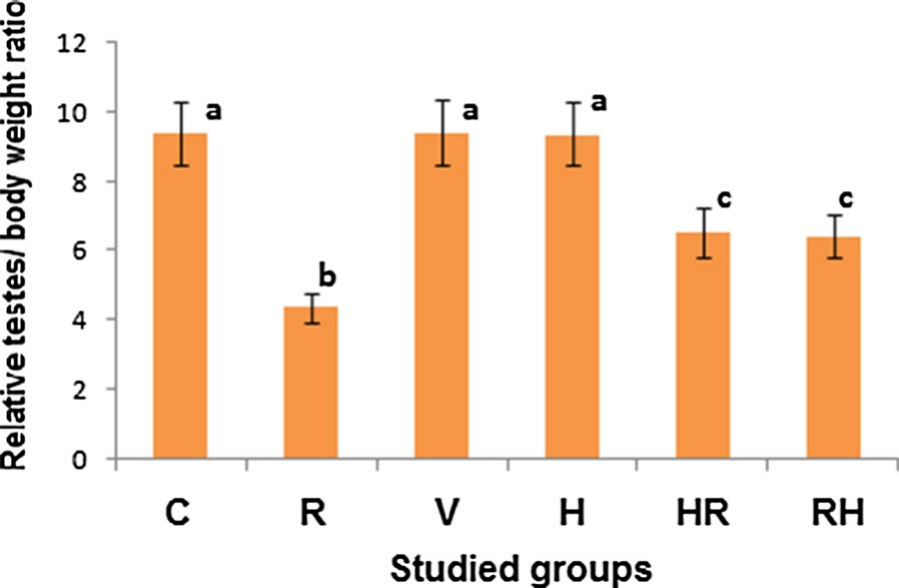
Relative testes/body weight ratio of all different studied groups. The values are expressed as mean ± SD. Number of rats in each group = 12. Values with different superscripts within the same column are statistically significant between groups at *p* < 0.05. C group: Control group; R group: Rats were irradiated with γ-radiation (total dose: 8 Gy); H group: Rats were administered with 200 mg of hesperidin (H) kg^−1^ body mass (b.m.), since H was dissolved in 1 ml of 99.6% DMSO; V group: Rats were administered with 1 ml of 99.6% DMSO kg^−1^ (b.m); HR group: Rats were treated with the same dose of H before their irradiation with the same dose of γ-radiation; RH group: Rats were irradiated with the same dose of γ-radiation then they treated with the same dose of H

**Fig. 4:**
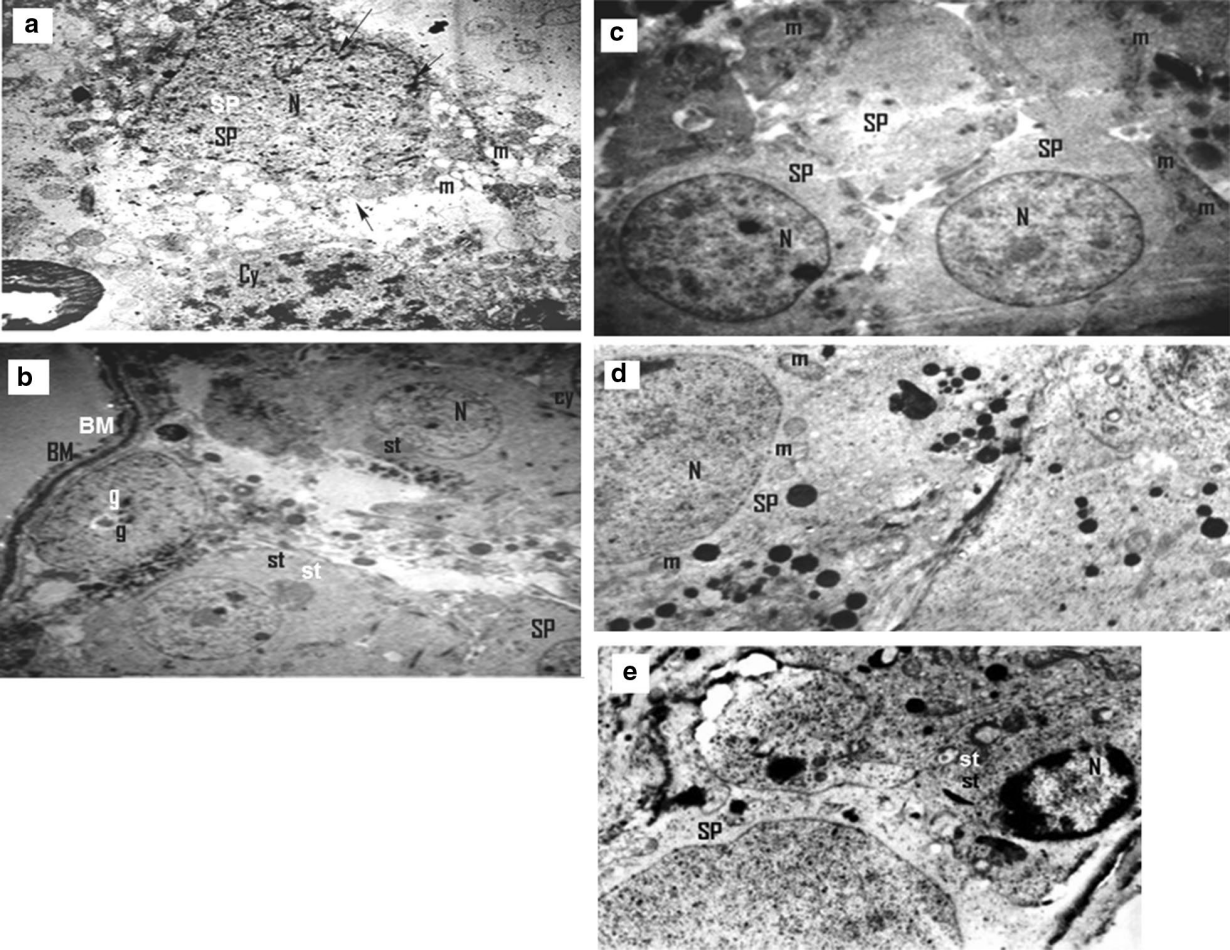
Microscopic examination of testes tissues of different studied groups. **a** Section in the testis of the control rats (or rats administered DMSO) showing the thin basement membrane (BM) surrounding the seminiferous tubule. Sertoli cells (St) with indented euchromatic nuclei (N), spermatid (Sp) and spermatocyte (Cy). Spermatogonia (g) with ovoid nucleus (X-6000); **b** Section in the testis of irradiated rats showing highly degenerated (SP) with deteriorated cytoplasm and blebbing of nuclear membrane (*arrow*), mitochondria appear as empty vesicles and (Cy) with nuclei contain clumps of heterochromatin (X-8000); **c** Section in the testis of rats receiving hesperidin (H) showing transverse sections of (SP) with large euchromatic nuclei and their cytoplasm contains peripherally located mitochondria (X-6000); **d** Section in the testis of rats receiving H before γ-radiation showing regeneration of (SP) with healthy mitochondria (m) and (N) (X-3600); **e** Section in the testis of rats receiving H after γ-radiation showing relatively regeneration of (SP) with still damaged sertoli cells which contained condensed heterochromatic (N) (X-3600). Number of rats in each group was 12

**Fig. 5:**
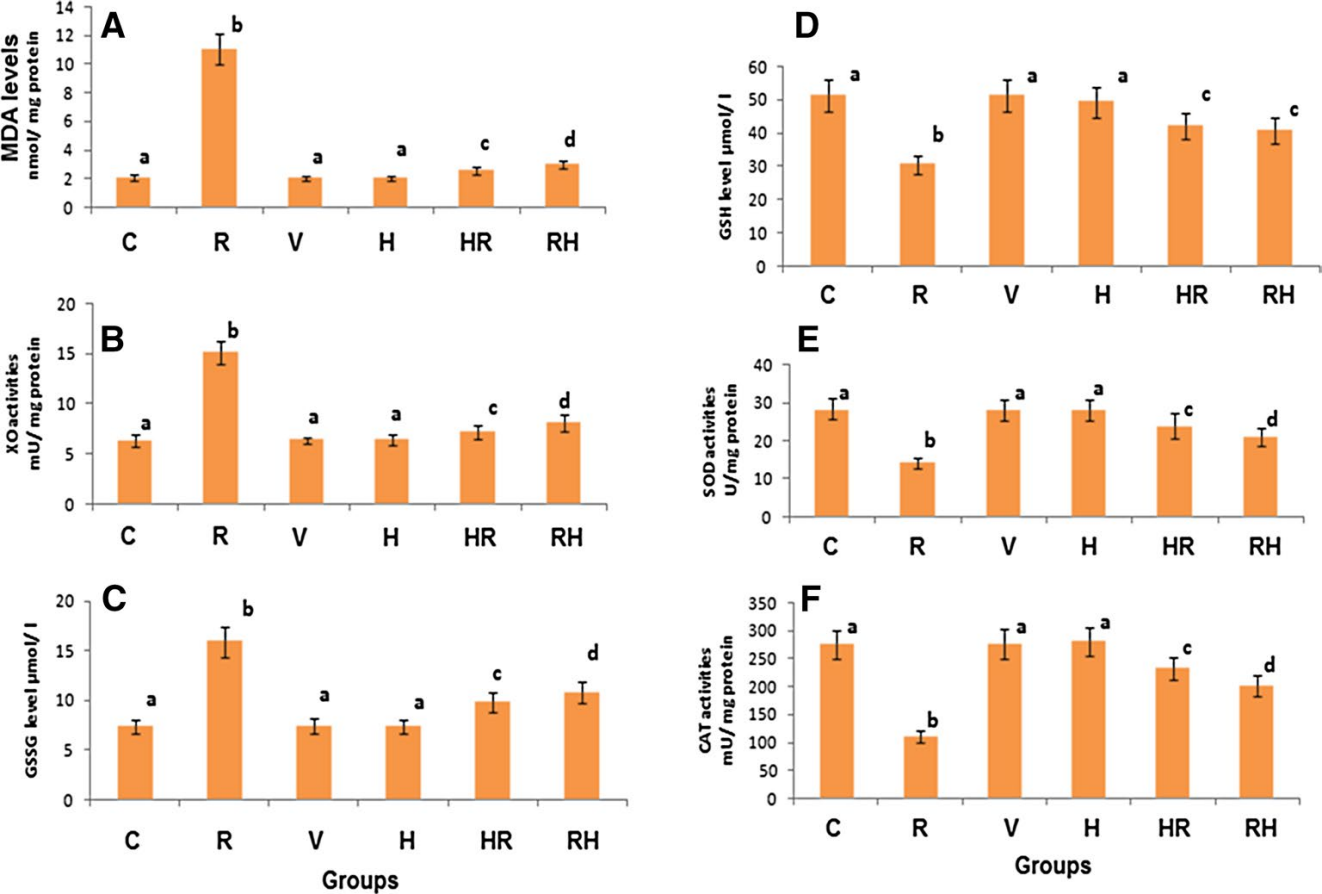
Treatment effects on the levels of lipid peroxidation and antioxidant parameters. **A** MDA level (an end product of lipid peroxidation); **B** XO activity; **C** nuclear oxidized glutathione (N-GSSG); **D** nuclear reduced glutathione (N-GSH); **E** SOD activity and **F** CAT activity. The values are expressed as mean ± SD. Number of rats in each group was 12. Values with *different superscripts* within the same column are statistically significant between groups at *p* ≤ 0.05. C group: Control group; R group: Rats were irradiated with γ-radiation (total dose: 8 Gy); H group: Rats were administered with 200 mg of hesperidin (H) kg^−1^ body mass (b.m.), since H was dissolved in 1 ml of 99.6% DMSO; V group: Rats were administered with 1 ml of 99.6% DMSO kg^−1^ (b.m); HR group: Rats were treated with the same dose of H before their irradiation with the same dose of γ-radiation; RH group: Rats were irradiated with the same dose of γ-radiation then they treated with the same dose of H

In the present study, exposure of whole body of rats to 8 Gy (R group) showed a significant decrease (*p* < 0.05) in the relative testis/body weight ratio by about 53% as compared to the C group (Fig. 3). The biochemical data showed that γ-radiation induced significant elevations in the levels of DNAF (*p* < 0.05), Ca^2+^ (*p* < 0.05) and NO (*p* < 0.05) in testis mitochondria by about 143, 186 and 143%, respectively, as compared to C group (Table 1). Also, γ-radiation caused significant elevations (*p* < 0.05) in MDA level and XO activity and oxidized glutathione (GSSG) level by about 430, 140 and 116%, respectively, concomitant with significant decreases (*p* < 0.05) in GSH level and SOD and CAT activities by about 41, 50 and 60%, respectively, as compared to the C group (Fig. 5A–F). Otherwise, γ-radiation caused significant increases (*p* < 0.05) in the levels of triacylglycerol (TG), total cholesterol (TC), very low density lipoprotein cholesterol (VLDL-C) in serum by about 75, 31 and 75%, respectively, with a significant decrease (*p* < 0.05) in HDL-C level by about 49% as compared to the C group (Table 2). Testicular high density lipoprotein cholesterol (HDL-C) and low density lipoprotein cholesterol (LDL-C) levels were significantly increased (*p* < 0.05) by about 39 and 319%, respectively (Table 2). On the other hand, exposure to γ-radiation (R group) revealed degeneration of sertoli cells, swelling mitochondria appearing as empty vesicles, highly degenerated spermatids and cluster of spermatids with a characterized chromosomal “cap”, deteriorated cytoplasm and cytoplasmic tag, blebbing of cells, abnormal nuclei containing condensed chromatin (Fig. 4a).

**Table 1:** Effect of hesperidin on DNAF, Ca^2+^and NO in rat testis of different studied groups

Groups	DNAF as % in testes homogenate	Ca^2+^ (mg ml^−1^)	NO (µmol l^−1^)
		In mitochondrial matrix	
C	5.13 ± 0.50^a^	0.66 ± 0.066^a^	26.40 ± 2.77^a^
R	12.46 ± 1.20^b^	1.89 ± 0.19^b^	64.20 ± 7.45^b^
V	5.15 ± 0.25^a^	0.64 ± 0.067^a^	26.20 ± 2.60^a^
H	5.38 ± 0.51^a^	0.69 ± 0.074^a^	24.30 ± 2.24^a^
HR	6.10 ± 0.60^c^	0.97 ± 0.10^c^	31.50 ± 3.66^c^
RH	6.29 ± 0.60^c^	0.99 ± 0.10^c^	37.40 ± 3.53^d^
LSD	0.705	0.27	5.1
F value	211.6	230.8	166.5

The values are expressed as mean ± SD. Values with different superscripts within the same column are statistically significant at *p* < 0.05. Number of rats in each group was 12. C group: Control group; R group: Rats were irradiated with γ-radiation (total dose: 8 Gy); H group: Rats were administered with 200 mg of hesperidin (H) kg^−1^ body mass (b.m.), since H was dissolved in 1 ml of 99.6% DMSO; V group: Rats were administered with 1 ml of 99.6% DMSO kg^−1^ (b.m); HR group: Rats were treated with the same dose of H before their irradiation with the same dose of γ-radiation; RH group: Rats were irradiated with the same dose of γ-radiation then they treated with the same dose of H

**Table 2:** Effect of H administration on lipid profile in rat testis and serum of different studied groups

Groups	HDL-C	LDL-C	HDL-C	LDL-C	TC	TG	VLDL-C
	In testes homogenates (mg g^−1^ wet tissue)		In serum (mg dl^−1^)				
C	10.16 ± 1.1^a^	5.03 ± 0.5^a^	14.52 ± 1.4^a^	53.42 ± 5.1^a^	95.03 ± 9.1^a^	53.75 ± 5.1^a^	10.75 ± 1.0^a^
R	14.11 ± 1.1^b^	21.10 ± 2.0^b^	7.44 ± 0.7^b^	88.20 ± 8.4^b^	124.20 ± 11.8^b^	94.13 ± 8.9^b^	18.83 ± 1.8^b^
V	10.16 ± 1.1^a^	5.03 ± 0.5^a^	14.30 ± 1.5^a^	53.40 ± 5.2^a^	95.10 ± 9.1^a^	53.60 ± 5.0^a^	10.77 ± 1.0^a^
H	10.32 ± 1.2^a^	5.20 ± 0.5^a^	13.53 ± 1.3^a^	50.20 ± 4.8^a^	92.83 ± 8.8^a^	54.10 ± 5.2^a^	10.82 ± 1.0^a^
HR	12.24 ± 1.2^c^	7.40 ± 0.7^c^	10.90 ± 1.0^c^	63.54 ± 6.1^c^	106.10 ± 10.9^c^	62.67 ± 6.0^c^	12.95 ± 1.2^c^
RH	11.75 ± 1.1^c^	7.45 ± 0.7^c^	10.43 ± 1.0^c^	67.53 ± 6.4^c^	105.83 ± 9.7^c^	66.63 ± 6.4^c^	13.33 ± 1.3^c^
LSD	1.43	2.2	0.9975	10.125	10.79	8.57	2.14
F value	21.76	487.85	75.81	68.46	18.02	78.05	77.89

The values are expressed as mean ± SD. Number of rats in each group was 12. Values with different superscripts within the same column are statistically significant between groups at *p* < 0.05. C group: Control group; R group: Rats were irradiated with γ-radiation (total dose: 8 Gy); H group: Rats were administered with 200 mg of hesperidin (H) kg^−1^ body mass (b.m.), since H was dissolved in 1 ml of 99.6% DMSO; V group: Rats were administered with 1 ml of 99.6% DMSO kg^−1^ (b.m); HR group: Rats were treated with the same dose of H before their irradiation with the same dose of γ-radiation; RH group: Rats were irradiated with the same dose of γ-radiation then they treated with the same dose of H

The histological examination of rat testes which administered with DMSO (V group) appeared normal with no changes in the ultra structure configuration when compared with the C group (Fig. 4b). In addition, V group revealed non significant changes (*p* > 0.05) in the relative testis/body weight ratio (Fig. 3) and all biochemical parameters as compared with the C group (Fig. 5A–F).

### Effect of H on testes injury induced by γ-radiation

Administration of H before or after γ-radiation (HR and RH groups, respectively) showed a significant amelioration of the γ-radiation-induced damage in the ultra structure of testicular tissues (Fig. 4d, e). Also, the figure showed a regeneration of spermatids with healthy nuclei and mitochondria. HR and RH groups showed significant increases (*p* < 0.05) in the relative testis/body weight ratio by about 49 and 47%, respectively, as compared to the R group (Fig. 3). Also, HR and RH groups showed significant decreases (*p* < 0.05) in the levels of DNAF, Ca^2+^ and NO (by about 51, 49 and 51%, respectively, in the case of HR group and by 49.50, 48.00 and 42.00%, respectively, in the case of RH group) as shown in Table 1. Moreover, XO activity, MDA and GSSG levels were decreased significantly (*p* < 0.05) by about 52, 76 and 38.3%, respectively, for HR group and by 46, 73 and 32%, respectively, for RH group as compared to R groups (Fig. 5A–C). Treatment with HR caused significant increases (*p* < 0.05) in the levels of SOD and CAT activities and GSH by about 68, 109 and 38%, respectively, as compared to R groups. Also, RH treatment increased significantly (*p* < 0.05) the activities of SOD and CAT and GSH level by about 48, 82, and 34%, respectively, (Fig. 5D–F). HR and RH groups showed significant increases (*p* < 0.05) in the testicular nuclear GSH/GSSG ratio by about 122 and 96%, respectively, as compared to R group.

HR and RH groups showed significant decreases (*p* < 0.05) in the levels of TG (by about 33 and 29%, respectively), and VLDL-C (by about 31 and 29%, respectively) and TC (15% for both treatments) in serum as compared to R group (Table 2). The testicular HDL-C levels in HR and RH groups were decreased significantly (*p* < 0.05) by about 13 and 17%, respectively, as compared to R group (Table 2).

### Effect of H on healthy rats

Histological examination of testes of rats which administered with H only (H group) appeared normal with no changes in the ultrastructure configuration (Fig. 4c). H group exhibited a non-significant decrease (*p* > 0.05) in relative testes/body weight ratio by about 0.10% as compared to the C group (Fig. 3). Also it showed a non-significant elevation (*p* > 0.05) in the levels of DNAF, MDA, Ca^2+^ beside CAT and XO activities by about 4.90, 1.60, 4.60, 2.00 and 2.00%, respectively, as compared to the C group (Table 1). In contrast, NO level, SOD activity, GSH and GSSG levels were decreased non-significantly (*p* > 0.05) by about 8%, 1.00, 4.00 and 0.50%, respectively (Table 1 and Fig. 4). This treatment exhibited a non-significant decrease (*p* > 0.05) in GSH/GSSG ratio by about 3.40% as compared to the C group. The H group showed non-significant decreases (*p* > 0.05) in the levels of TC, HDL-C, LDL-C, VLDL-C by about 2.32, 7.00, 6.00, 0.70%, respectively, However, serum TG was increased non-significantly (*p* > 0.05) by 0.65% as compared to the C group (Table 2). In addition administration of H showed non-significant increases (*p* > 0.05) in testicular LDL-C and HDL-C levels by about 3.40 and 1.60% as compared to the C group (Table 2).

## Discussion

Histological examination of testicular tissues of irradiated rats with γ-radiation in a total dose of 8 Gy revealed degeneration of sertoli cells, swelling mitochondria which appeared as empty vesicles, highly degenerated spermatids with a characterized chromosomal “cap”, deteriorated cytoplasm with cytoplasmic tag and blebbing of cells containing abnormal nuclei with condensed chromatin. Also, the biochemical results showed that γ-radiation increased MDA, NO, DNAF, Ca^2+^ and GSSG levels as well as XO activity in testes, while GSH level and the activities of SOD and CAT were decreased as compared to the C group. This indicates that γ-radiation induced LPO and apoptosis in testicular tissues leading to a decrease in the relative testis/body weight ratio. The increase in DNAF may be also due to the effect of free radicals and this is concurring with the elevation of MDA level and XO. These results agree with the previous studies which reported that IR induced DNAF, activates p53, increases Bax (pro-apoptotic) and decreases Bcl2 protein expression (antiapoptotic), activates procaspases and stimulates apoptosis [11, 12].

It has been reported that intracellular calcium homeostasis is important for cell survival. In contrast, increase in mitochondrial calcium induces opening of permeability transition pore, mitochondrial dysfunction and apoptosis [13]. Consequently, the elevation in mitochondrial calcium and elevation in XO activity and MDA level, after radiation in this study, are involved in cell death; they might be involved in degeneration of testis. The increase in XO activity may be due to the increase of Ca^2+^ concentration since Ca^2+^ overload activates the protease calpain which converts xanthine dehydrogenase (XDH) to XO [14]. The detrimental effects of IR are associated with alteration in the xanthine oxidoreductase (XOR) system through the conversion of XDH into XO. The XOR system consists of two inter-convertible forms, XO and XDH, the later account about 90% of the total activity of XOR and has no role in the initiation of oxidative damage in the cells. However, in some pathological conditions, XDH is converted to XO leading to increased production of ${\text{O}}_{2}^{ \cdot - }$ radicals which converted into H_2_O_2_ and finally highly reactive ${}^{ \cdot }{\text{OH}}$, that initiate the LPO chain reaction. On the other hand, the ${\text{O}}_{2}^{ \cdot - }$ radicals may react with NO forming peroxynitrite anion (ONOO^−^) causing damage of DNA and activation of nuclear poly-ADP-ribose polymerase (PARP-1). PARP-1 catalyzes the hydrolysis of NAD^+^ which results in cellular energy failure and necrotic cell death [15]. Furthermore, mitochondrial NO^·^ accumulation leads to mitochondrial depolarization and release of mitochondrial cytochrome c into the cytosol [16].

The elevation of MDA level may be due to the effect of ${\text{O}}_{2}^{ \cdot - }$, H_2_O_2_, ^·^OH and ONOO^−^ radicals which interact with polyunsaturated fatty acids in the phospholipids of cell membrane inducing LPO in testis tissues [17, 18]. The depletion of GSH level may be owed to either its utilization in the detoxification of H_2_O_2_ or reaction with NO or ONOO^−^ to form S-nitrosoglutathione [19]. Also, increased demand of this tripeptide for lipid hydroperoxide metabolism by GPx led to its depletion [20]. Furthermore, DNAF impair the normal synthesis of GSH [21]. As shown from our results, the reduction in GSH level agrees with the elevation in GSSG level. The decrease in the activities of SOD and CAT in testes homogenate may be due to their denaturation by γ-radiation and free radicals. In addition γ-radiation induced cell membrane damage and this led to release these enzymes into the blood stream [22, 23]. Elevation of TG, TC, LDL-C and VLDL-C levels in serum and reduction of HDL-C level may be due to release of fats through the damaged cell membrane into the circulation. The increase of cholesterol level may be due to decrease in its utilization for synthesis of higher substances. The increase in serum TG level may be to the inhibition of lipoprotein lipase [24]. Also, the synthesis of cholesterol and TG in liver was increased [25]. The elevation of serum LDL-C level may be due to the damage induced by γ-radiation to the receptors on the surface of many cells in the body that prevents the ingestion of LDL-C by endocytosis [25]. On the other hand, γ-radiation caused significant elevations in the levels of testicular LDL-C, HDL-C and the LDL-C/HDL-C ratios. This indicates that γ-radiation induced LPO and apoptosis in testicular tissue. These results agree with the previous studies which reported that the oxidized low-density lipoprotein cholesterol changes Bcl-2 family proteins and activates Fas pathway leading to apoptosis [26].

Conversely, the electron micrograph of a section in the testis of rats administered with H before exposure to γ-radiation (HR) showed regeneration of spermatids with healthy mitochondria and nucleus. Treatment with H after radiation (RH) showed relative regeneration of spermatids with still damaged sertoli cells containing condensed heterochromatic nucleus. This indicates that H has protective and therapeutic roles against the dangerous effect of γ-radiation. Also, administration with H before γ-radiation gave better results than the treatment with H after exposure to γ-radiation. These results agree with the biochemical results which showed that H administration either before or after γ-radiation reduced the apoptosis induced by γ-radiation since the levels of DNAF and Ca^2+^ were decreased as compared with R group. Additionally, the levels of MDA, NO, and GSSG and XO activity were decreased, while GSH and GSH/GSSG ratio levels and the activities of SOD and CAT were increased as compared to R group. This indicates that H decreased OS and LPO leading to the reduction of testis injuries. Moreover lipid profile in serum and testis was improved since HDL-C, LDL-C and LDL-C/HDL-C ratio levels were decreased as compared to R group. Also, the levels of TG, TC and VLDL-C in serum were decreased. Therefore, the relative testis/body weight ratio was increased as compared to R group. The modulator role of H on OS may be attributed to its antioxidant and free radicals scavenging activities [27]. The previous studies confirmed that H can function as metal chelators and reducing agents, scavengers of ROS, chain-breaking antioxidants, quenchers of the formation of singlet oxygen, and protectors of ascorbic acid [28]. Also, H inhibits caspase-3 activity, prevents the decrease of Bcl-2 protein and the increase of Bax protein [29]. The results showed that the administration of DMSO (V group) caused a non significant change (increase or decrease) in some studied parameters as compared with the C group. Also, the histological examination of testis after DMSO administration showed normal with no changes in the ultrastructure configuration. This indicates that DMSO (dose: 1 ml of 99.6%/kg b.m.) had no side effect. On the other hand, it has been reported that the regular consumption of flavonoid-containing foods can reduce the risk of diseases [30]. In addition, the results of the present study showed that the administration of rats with H which dissolved in DMSO (H group) caused non significant (*p* > 0.05) changes in some biochemical parameters and relative testis/body weight ratios as compared with the C group and this may be due to the effect of DMSO. In contrast, the histological examination of testis of H group was normal with no changes in the ultra structure configuration. This indicates that administration of H for a short period does not cause side effects. These results agree with previous studies which showed no signs of toxicity have been observed with the normal intake of hesperidin or related compounds [31].

## Conclusion

1. Exposure of rats to γ-radiation induced OS, LPO and apoptosis and testes injury.
2. Treatment of rats with H before or after γ-radiation improved the architecture of testes since it decreased LPO, apoptosis and testes injury (i.e. H has antioxidant and antiapoptotic activities).
3. Protection is more effective when H is given before rather than after exposure.

## Methods

### Chemicals

H, xanthine solution, xanthine sodium salt, H_2_O_2_, thiobarbituric acid (TBA),diphenylamine, pyrogallol, standard SOD, GSH, GSSG and all chemicals were obtained from Sigma-Aldrich, St Louis, MO, USA.

### Animals

One hundred and two male Albino rats Sprague–Dawley (8 ± 2 weeks old; 80 ± 10 g body weight) were obtained. All rats were examined for health status and their room was designed to maintain the temperature at 25 °C, relative humidity at approximately 50% and 12 h light/dark photoperiod for 2 weeks prior to experimentation. The animals were then housed in stainless-steel cages, given standard diet and water ad libitum throughout the study and observed daily for abnormal signs. After acclimatization, 30 rats were used for determination of γ-radiation dose that induced testis injury (preliminary experiment), while 72 rats were used for determination of the effect of H on rat testis injury induced by γ-radiation.

### Radiation exposure

Whole body gamma irradiation of rats was performed with a 137 Cesium source in a Gamma cell 40 (Atomic Energy of Canada Ltd, Ottawa, Ontario, Canada). Animals were placed in the specially designed tray and received a definite dose of Gy delivered in four fractions at one day of interval at a dose rate of 0.5 Gy min^−1^.

### Preliminary experiment for determination of γ-radiation dose that induced testis injury

Rats were divided randomly into six groups, five rats each. Group 1 (control) did not receive γ-radiation while the rest five groups were subjected to whole body γ-radiation (groups 2–6: 2, 4, 6, 8 and 10 Gy, respectively), installed as 2 Gy each other day. At the end of the radiation periods the rats were fasted over night prior to anaesthesia and sacrificing. Testes were excised from animals and divided into two parts. The first part was used to study the transmission electron microscopy. The second part was washed with cold 0.1 M sodium phosphate buffer saline, pH 7.4, containing 0.16 mg ml^−1^ heparin and weighed. Then the testes tissues were homogenized in 5 volume (weight/volume “w/v”) of cold 0.05 M sodium phosphate containing 1 mM ethylenediamine-tetraacetic acid (EDTA) pH 7.4, using a glass-Teflon homogenizer. The homogenate was centrifuged at 10,000×*g* for 15 min at 4 °C, and the supernatant was stored at −20 °C till used for the determination of DNAF using diphenylamine [33].

### Effect of H on rat testis injury

Rats were divided into six groups of 12 rats each. C group: rats did not receive any treatment. R group: rats whole bodies were exposed to γ-radiation installed as 2 Gy each other day up to a total dose of 8 Gy (according to the results of the preliminary experiment). H group: rats were administered orally (using oral gavage) with H [dose: 200 mg kg^−1^ body mass (b.m.) since H was dissolved in 1.0 ml of 99.6% DMSO] for 7 successive days [34]. V group: rats were administered orally with DMSO to show its effect (dose: 1.0 ml of 99.6% DMSO kg^−1^ b.m.) for 7 successive days. HR group: rats were administered orally with H (200 mg kg^−1^ b.m.) for 7 successive days, then, at 8th day rats were whole body subjected to γ-radiation as mentioned before in R group. RH group: rats were irradiated with γ-radiation as mentioned before in R group, then at 8th day rats were administered with H as mentioned before in H group. At the end of the experimental periods (at 8th day for groups C, H and V and at 14th day for groups HR and RH), the rats were fasted over night prior to anaesthesia and sacrificing. Testes were excised from animals and divided into three parts.

The first part was examined by electron microscopy. The second part was washed with 0.1 M sodium phosphate buffer saline, pH 7.4, and then homogenized in 5 volumes (w/v) of cold 0.05 M sodium phosphate containing 1 mM EDTA, pH 7.4. The homogenate was centrifuged at 10,000×*g* for 15 min at 4 °C, and the supernatant was used for determination of XO, CAT, SOD, MDA, DNAF, TP, high density lipoprotein cholesterol (HDL-C) and low density lipoprotein cholesterol (LDL-C). The third part of testes was used for preparation of nuclear matrix for determination of GSH beside mitochondrial matrix for determination of NO and Ca^2+^.

### Preparation of nuclear matrix and mitochondrial matrix

At first nuclei and mitochondria were isolated [35] since the testes were weighed, minced, and homogenized using Teflon-glass homogenizer in 10 volumes (w/v) of 50 mM Tris HCl buffer containing 0.25 M sucrose, 25 mM KCl and 5 mM MgCl_2_ (pH 7.4). The homogenate was centrifuged at 700×*g* for 10 min since the pellet was separated as the nuclear fraction and the supernatant was re-centrifuged at 10,000×*g* for 10 min, and the pellet in this case was taken as the mitochondrial fraction and the supernatant was discarded. Then, nuclear and mitochondrial matrices were prepared according to the procedure of Robinson [36]. In brief, nuclear and mitochondrial fractions separately were homogenized in 12 volumes of 8 M HCl, boiled gently on a hot plate for 5 min, cooled and centrifuged at 10,000×*g* for 10 min. The supernatants, nuclear and mitochondrial matrices, respectively, were separated and kept at −20 °C until used, while the pellets were discarded. Nuclear matrix was used for determination of total glutathione, GSSG and GSH but NO and Ca^2+^ was determined in mitochondrial matrix.

Blood samples were taken from rats under diethyl ether anesthesia by heart puncture. Unheparinized blood samples were centrifuged at 1000×*g* for 20 min and sera were stored at −20 °C until used for determination of TC, HDL-C, LDL-C, VLDL-C and TG.

### Biochemical analysis

#### MDA level

It was determined in testes homogenates [37], since MDA reacts with TBA in acidic medium giving MDA-TBA adducts exhibiting a pink colored which measured at 532 nm using a T60 UV/VIS spectrophotometer (PG instruments, London, UK). MDA is defined as nmole mg^−1^ protein.

#### Xanthine oxidase (XO, EC: 1.17.3.2) activity

It is a form of XOR. XO was determined in testes homogenates according to the method of Bergmeyer et al. [38] in which the rate of formation of uric acid was followed at 290 nm using spectrophotometer. The enzyme catalyzes the conversion of xanthine into uric acid as follows:

$${\text{Xanthine}} + {\text{H}}_{2} {\text{O}} + {\text{O}}_{2} \mathop {\longrightarrow}\limits^{{{\text{Xanthine}}\;{\text{oxidase}}}}\;{\text{Uric}}\;{\text{Acid}} + {\text{H}}_{2} {\text{O}}_{2}$$

One unit of XO activity is defined as amount of enzyme required to catalyze the formation of 1 µmol of uric acid per minute at 25 °C and pH 7.5. The enzyme activity is expressed as U mg^−1^ protein.

#### Concentration of NO

It was determined in mitochondrial matrix [39]. In this method NO was determined as total nitrite concentration by converting nitrate to nitrite in the presence of cadmium as reducing agent as shown from the following reactions. NO concentration was calculated as μmol l^−1^.

$$\begin{aligned} & {\text{NO}} + {\text{O}}_{2}^{ - \cdot } \to {\text{ONOO}}^{ - } \mathop{\longrightarrow}\limits^{{{{\text{H}}} + }}{\text{NO}}_{3}^{ - } + {\text{H}}^{ + } \mathop{\longrightarrow}\limits^{{\text{Cd}}}{\text{NO}}_{2}^{ - } \\ & 2{\text{NO}} + {\text{O}}_{2} \to {\text{N}}_{2} {\text{O}}_{4} \mathop{\longrightarrow}\limits^{{{{\text{H}}_{2} {\text{O}}}}}{\text{NO}}_{2}^{ - } + {\text{NO}}_{3}^{ - } + 2{\text{H}}^{ + } \mathop{\longrightarrow}\limits^{{\text{Cd}}}{\text{NO}}_{2}^{ - } \\ &{\text{NO}} + {\text{NO}}_{2}^{ - } \to {\text{N}}_{2} {\text{O}}_{3} \mathop{\longrightarrow}\limits^{{{{\text{H}}_{2} {\text{O}}}}}2{\text{NO}}_{2}^{ - } + 2{\text{H}}^{ + } \\ \end{aligned}$$

#### GSH and GSSG levels

Total glutathione in nuclear matrix was determined according to the method of Beutler et al. [40] using 5, 5′-dithiobis-2-nitrobenzoic acid (DTNB), Ellman’s reagent, through the following reactions:

$$ \begin{aligned} &2{\text{GSH}} + {\text{DTNB}} \to {\text{GSSG}} + 2 {\text{TNB}} \\  &{\text{GSSG + NADPH + H}}^{ + } \mathop{\longrightarrow}\limits^{{\text{GR}}}2{\text{GSH}} + {\text{NADP}}+  \\&{\text{DTNB + NADPH + H}}^{ + } \mathop{\longrightarrow}\limits^{{\text{GSH - CSSG GR}}}2{\text{TNB}} + {\text{NADP}}^{ + } \\&\qquad \qquad \qquad \qquad \qquad \qquad({\text{Yellow}}\;{\text{color}}) \\ \end{aligned} $$

The yellow color of TNB was measured at 405 or 414 nm using spectrophotometer.

The concentration of GSSG was determined by derivatization of GSH by 4-vinylpyridine since the absorbance was measured at 412 nm using spectrophotometer [41]. Then GSH concentration was calculated from the following equation:

$${\text{GSH}}\;{\text{concentration}} = {\text{Total}}\;{\text{glutathione}}\;{\text{concentration}} - {\text{GSSG}}\;{\text{concentration}}$$

#### Superoxide dismutase (SOD, EC: 1.15.1.1)

Cu–Zn–SOD activity was determined in testes homogenates according to the methods of Marklund and Marklund [42]. SOD plays an important role in the removal of O_2_^−·^ radical according to the following equation:

$${\text{O}}_{2}^{ - \cdot } + {\text{O}}_{2}^{ - \cdot } + 2{\text{H}}^{ + } \mathop{\longrightarrow}\limits^{{\text{SOD}}}{\text{H}}_{2} {\text{O}}_{2} + {\text{O}}_{2}$$

One unit of SOD activity is defined as the amount of enzyme which inhibits the rate of autoxidation of pyrogallol by 50% and expressed as IU mg^−1^ protein.

#### Catalase (CAT, EC: 1.11.1.6) activity

It catalyzes the decomposition of H_2_O_2_ into water and oxygen as in the following reaction:

$$2{\text{H}}_{2} {\text{O}}_{2} \mathop{\longrightarrow}\limits^{{\text{catalase}}}2{\text{H}}_{2} {\text{O}} + {\text{O}}_{2}$$

CAT was determined in testes homogenates according to the methods of Aebi [43] since the disappearance of peroxide was followed at 240 nm using spectrophotometer. One unit of CAT activity is defined as the amount of enzyme required to decompose one µmole of H_2_O_2_ per min at 25 °C and pH 7. The specific activity of CAT is expressed as U mg^−1^ protein.

#### DNAF

The percentage of DNAF in testes homogenates was determined using diphenylamine since the color was read at 578 nm with ELISA reader; a blank was sited to zero [33, 44].

#### Calcium concentration

It was determined in mitochondrial matrix by Atomic Absorption Spectrophotometer (Perkin-Elmer, Model 2380, USA) [36].

#### Determination of TP

It was determined in serum and testes homogenates according to the method of Lowry et al. [45].

#### Assay of lipid profile

The levels of TG, TC, LDL-C, HDL-C and VLDL-C in serum and testes homogenates were determined [46–49].

#### Transmission electron microscopy

Testes specimens (cubic specimens 1 mm in edge) were prepared for electron microscopy according to the method of Weakley [50] which includes the following processes: (1) Fixation: Double fixation technique was used since 2% glutaraldehyde solution and 2% osmic tetroxide solution were utilized; (2) Dehydration: Testis specimens were dehydrated through a grade ethanol series 30% to absolute ethanol; (3) Embedding: Epoxy resins (Sppur Kit) were used for embedding; (4) Polymerization: Epoxy-resin-embedded capsules were left in an oven at 60 °C for 18 h; (5) Cutting: Ultra-thin sections were cut with 6 mm glass knives to get sections 700 Å in thickness using LKB Ulteratome III; (6) Double staining: Ultra thin sections were usually contrasted with uranyl acetate then lead citrate and (7) Examination and photography: Ultra-thin sections were viewed, examined and photographed with JEOL (JEM 100CX) Transmission Electron Microscope.

### Statistical analysis

The data were given as individual values and as means ± standard deviation (SD) for animals in each group. Comparisons between the means of various treatment groups were analyzed using Least Significant Difference (LSD) test for each parameter tested. Differences were considered significant at *p* < 0.05. All statistical analyses were performed using the statistical software SPSS, IBM, version 11.5.

## Abbreviations

CAT: catalase
COX-2: cyclooxygenase-2
DMSO: dimethylsulfoxide
DNAF: DNA-fragmentation
EDTA: ethylenediamine-tetraacetic acid
GPx: glutathione peroxidase
GS^**·**^: thiyl radicals
GSH: reduced glutathione
GSSG: oxidized glutathione
γ: gamma
H: hesperidin
H_2_O_2_: hydrogen peroxide
HDL-C: high density lipoprotein cholesterol
HR: H before Γ-radiation
IR: ionizing radiation
LDL-C: low density lipoprotein cholesterol
LPO: lipid peroxidation
MDA: malondialdehyde
NO: nitric oxide
NO^**·**^: nitric oxide radical
^**·**^OH: hydroxyl radicals
O_2_^−**·**^: superoxide anions
ONOO^–^: peroxynitrite anion
OS: oxidative stress
PARP-1: poly-ADP-ribose polymerase
RH: H after Γ-radiation
ROS: reactive oxygen species
SOD: superoxide dismutase
TBA: thiobarbituric acid
TC: total cholesterol
TG: triacylglycerol
TP: total protein
V: vehicle
VLDL-C: very low density lipoprotein cholesterol
XDH: xanthine dehydrogenase
XO: xanthine oxidase
XOR: xanthine oxidoreductase
